# miR-143 is implicated in growth plate injury by targeting IHH in precartilaginous stem cells

**DOI:** 10.7150/ijms.46474

**Published:** 2021-03-03

**Authors:** Fu-Yong Zhang, Yun-Fang Zhen, Zhi-Xiong Guo, Jin Dai, Lun-Qing Zhu, Pei-Rong Liang, Guang-Hao Su, Wen-Yan Zhang, Jian-Feng Fang, Quan-Wen Yuan, Feng Yao, Ya Liu, Yi Qiao, Ya Zhang, Wan-Liang Guo, Yao Liu, Xiao-Dong Wang

**Affiliations:** 1 Department of Orthopaedics, Children's Hospital of Soochow University, Suzhou, 215000, China.; 2 Department of Radiology, Children's Hospital of Soochow University, Suzhou, 215000, China.

**Keywords:** *miR-143*, precartilaginous stem cells, IHH

## Abstract

Precartilaginous stem cells (PCSCs) are able to initiate chondrocyte and bone development. The present study aimed to investigate the role of *miR-143* and the underlying mechanisms involved in PCSC proliferation. In a rat growth plate injury model, tissue from the injury site was collected and the expression of *miR-143* and its potential targets was determined. PCSCs were isolated from the rabbits' distal epiphyseal growth plate. Cell viability, DNA synthesis, and apoptosis were determined with MTT, BrdU, and flow cytometric analysis, respectively. Real time PCR and western blot were performed to detect the mRNA and protein expression of the indicated genes. Indian hedgehog (*IHH*) was identified as a target gene for *miR-143* with luciferase reporter assay. Decreased expression of *miR-143* and increased expression of *IHH* gene were observed in the growth plate after injury. *miR-143* mimics decreased cell viability and DNA synthesis and promoted apoptosis of PCSCs. Conversely, siRNA-mediated inhibition of *miR-143* led to increased growth and suppressed apoptosis of PCSCs. Transfection of *miR-143* decreased luciferase activity of wild-type *IHH* but had no effect when the 3'-UTR of *IHH* was mutated. Furthermore, the effect of *miR-143* overexpression was neutralized by overexpression of IHH. Our study showed that *miR-143* is involved in growth plate behavior and regulates PCSC growth by targeting *IHH*, suggesting that *miR-143* may serve as a novel target for PCSC-related diseases.

## Introduction

Many clinical conditions require regeneration or implantation of bone. Current therapeutic options are not sufficient to treat massive bone defects. Stem cell-based therapies have been used as an alternative to current solutions for bone-loss repair 1. Precartilaginous stem cells (PCSCs) are a type of newly identified adult stem cells that exist in the most peripheral layer of the epiphyseal organ with a perichondrial mesenchyme in embryonic limbs. Studies have shown that PCSCs play diverse roles in endochondral ossification, limb growth, cartilage development, and repair of damaged articular cartilage 2. However, the underlying mechanisms involved in the acquisition of PCSC properties have not been elucidated.

Hedgehog (Hh) signaling is critical for the regulation of bone development, homeostasis, and repair. Indian hedgehog (Ihh), a member of Hh proteins, is expressed in the prehypertrophic and hypertrophic chondrocytes in the growth plate. Activation of Ihh signaling synchronizes chondrogenesis and osteogenesis during endochondral ossification by promoting chondrocyte differentiation and proliferation, and specification of bone-forming osteoblasts 3,4. Overexpression of Ihh enhances chondrogenesis and osteogenesis from mesenchymal stem cells (MSCs) 5,6. In addition, Ihh-regulated chondrogenesis is associated with different pathways, such as Wnt/β-Catenin, parathyroid hormone-related peptide (PTHrP), and TGF-β/Smad pathways 7-10. These findings suggest that targeting Ihh may be a novel therapeutic approach for bone-related diseases.

MicroRNAs (miRNAs) are a group of small, noncoding RNAs. By post-transcriptional regulation of their target mRNAs through complementary binding to the 3' untranslated region (UTR), miRNAs play diverse roles in cellular behaviors, including cell proliferation, apoptosis, differentiation, and angiogenesis 7. Accumulating evidence indicates that miRNA expression is critical for cartilage differentiation and skeletal development. For example, *miR-26a* and *miR-125b* have been discovered to suppress osteoblastic differentiation, while *miR-210* and *miR-29b* activate osteoblastic differentiation 12-14. *miR-143*, a miRNA located in chromosome 5q33, was reported to suppress osteogenic differentiation 15. Recently, Tian et al have found that overexpression of *miR-143* suppressed cartilage differentiation of bone marrow mesenchymal stem cells (BMSCs) via targeting bone morphogenetic protein 2 (BMPR2) 16. Consistently, another study confirmed that *miR-143* inhibits osteogenic differentiation of dental pulp stem cells (DPSCs) by targeting tumor necrosis factor (TNF)-α 17. However, the effect of *miR-143* on PCSC physiology and the underlying mechanisms involved have not been reported yet. In the present study, we explored whether *miR-143* regulates PCSC growth in an IHH-dependent manner, aiming to provide a novel therapeutic target for bone-related diseases.

## Materials and Methods

### Animals and growth plate injury model

All animal experiments were performed according to protocols approved by the Animal Care and Use Committee of Children's Hospital of Soochow University. Healthy 3-week-old New Zealand white rabbits were purchased from Shanghai Laboratory Animals Center (Shanghai China). Animals were housed under a 12-h light/dark cycle with unlimited access to food and water. Twelve rabbits underwent experimental drill-hole growth plate injury in the proximal tibia of both hind legs. Following anesthesia, the growth plate was made accessible through a cortical window at the center of growth plate. Three days later, animals in control and injury model groups (n=6 in each group) were euthanized and the epiphyseal plate from the proximal tibiae was harvested for later experiments. All animal experimentation was conducted in accordance with accepted standards of humane animal care in accordance with the NIH guidelines.

### Cell culture

Rabbits were euthanized with a lethal dose of sodium phenobarbital. The epiphyseal plate from the proximal tibiae was harvested, washed three times with ice-cold PBS, cut into thin sections, and digested with trypsin and collagenase II. The cell suspension was filtered through a 200-mesh stainless steel filter to remove any undigested particles. Finally, cells were maintained in DMEM medium supplemented with 10% FBS, 100 U/mL ampicillin, and 100 U/mL streptomycin in a 37 ºC incubator with 5% CO_2_. Cells were passaged at 1:5 dilution at 30-60% confluency using trypsin. Cells from generation 2-4 were used for further experiments.

### Immunofluorescence

Purified PCSCs at a density of 5×10^5^ cells/well were plated in six-well-plates, fixed with 4% paraformaldehyde, and permeabilized with 0.2% Triton X-100. Cells were then incubated with anti-FGFR-3 (1:1000 dilution, Santa Cruz) at 4 ºC overnight. Cells were then incubated with a FITC-labeled secondary antibody (Cellular Signaling Tech, Shanghai, China) for 1 h at room temperature. Cells were then observed and images recorded under a fluorescence microscope (Olympus, Tokyo, Japan).

### Cell growth assay

Cell viability was measured with MTT assay (MD Millipore, Billerica, MA, USA) at different time points (24, 48, and 72 h after transfection). Briefly, cells were cultured overnight at 3×10^4^ cells/well in 96-well culture plates. Cells were incubated with 5 mg/mL MTT at 37 °C for 2 h. The absorbance for each well was measured at 490 nm using a scanning multi-well spectrometer (Bio-Tek instruments, Inc., Burlington, VT, USA).

### BrdU incorporation assay

Cell proliferation was estimated using the BrdU Labeling and Detection Kit III (Roche Molecular Biochemicals, Indianapolis, IN, USA) following the manufacturer's instruction. Briefly, cells were plated in 96-well plates at a density of 4000 cells/well. Twenty-four hours later, cells were incubated with BrdU at 37 °C for 4 h and washed three times with PBS, followed by incubation with anti-BrdU conjugated with peroxidase at 37 °C for 30 min. The absorbance for each well was measured at 450 nm using a scanning multi-well spectrometer (Bio-Tek instruments, Inc., Burlington, VT, USA).

### miRNAs transfection

miRNA transfection was performed using Lipofectamine 2000 reagents (ThermoFisher Scientific, Waltham, MA, USA) according to the manufacturer's instructions. Briefly, 2×10^5^ cells/well were plated in a 6-well plate overnight. After adding new medium, cells were transfected with miRNA mimics or inhibitors followed by downstream assays.

### Apoptosis assay

Annexin V‑FITC/PI staining was performed to quantify the percentage of apoptotic cells. Cells were stained using the Annexin V‑FITC Apoptosis Detection kit (Invitrogen, Inc., Carlsbad, CA, USA) according to the manufacturer's protocol. After being washed with PBS, cells were resuspended in binding buffer, incubated with Annexin V‑FITC and PI, and analyzed on a BD Accuri C6 Flow Cytometer (BD Biosciences, USA).

### RNA isolation and real-time PCR

Total RNA was extracted using TRIzol (Invitrogen, Carlsbad, CA) according to the manufacturer's instructions. For detection of mRNA expression, cDNA synthesis was performed from 1 µg total RNA using a SuperScript First-Standard Synthesis kit (Invitrogen Life Technologies). Real-time PCR was performed with an ABI PRISM 7900 Sequence Detection System (Applied Biosystems, Foster City, CA, USA). The following primers were used: IHH: forward: 5'- CAAGAAGCCCGGGATCTACA-3, reverse: 5'- GCTCGGGACTTTGTTGCTTG-3' and β-actin: forward 5'- GGCTGTGCTATCCCTGTACG -3', reverse 5'- AGGTAGTCAGTCAGGTCCCG -3'. Reactions were incubated in a 96-well optical plate at 95 °C for 10 min, followed by 40 cycles at 95 °C for 15 s, 60 °C for 45 s, and 72 °C for 1 min. Relative expression was calculated using the 2^-ΔΔCT^ method.

### Plasmid DNA transfection

Transfection was performed using the Lipofectamine 2000 Transfection reagent (Invitrogen) according to the manufacturer's protocol. The eukaryotic expression vector pcDNA3.1(+) (Invitrogen) was used to generate the IHH expression plasmid. The IHH genomic sequence was amplified with PCR, digested with EcoRI and BamHI (Promega, Madison, WI, USA), and subcloned into the pcDNA3.1(+) vector.

### Luciferase assay

Luciferase reporter assay was performed after transient co-transfection of the psiCHECK2-report vector (Ambion, Austin, TX, USA) containing the wild type or mutant 3'UTR of the human *IHH* gene, which includes the putative *miR-143* binding site, into HEK293T cells in 24-well plates using Lipofectamine 2000 (Invitrogen, Carlsbad, CA) according to the manufacturer's instructions. For luciferase reporter assay, 48 hours after transfection, luciferase activity was measured using the Dual-Luciferase Reporter Assay System (Promega). Luciferase activity was monitored with SpectraMax M5 (Molecular Devices, Sunnyvale, CA, USA). Experiments were performed in triplicates and repeated three times.

### Western blot

Protein samples were separated with SDS-PAGE electrophoresis and transferred to nitrocellulose membranes. Membranes were incubated with rabbit monoclonal antibodies against IHH (dilution 1:1000, Abcam, Cambridge, UK) and β-actin (dilution 1:1000, Abcam, Cambridge, UK) overnight. Membranes were then incubated with HRP-conjugated anti-rabbit secondary antibodies (Abcam, Cambridge, UK) at room temperature for 1 h. Proteins were detected using a Super Signal Enhanced Chemiluminescence kit (Pierce, Rockford, IL, USA).

### Statistical analysis

Data are expressed as mean ± standard deviation (SD). Differences between two groups were compared using the Student's t-test, and mean values of multiple groups were compared using one-way ANOVA and Bonferroni post hoc test. *P*<0.05 was considered statistically significant.

## Results

### *miR-143* is involved in the growth and DNA synthesis of PCSCs

**Figure 1:**
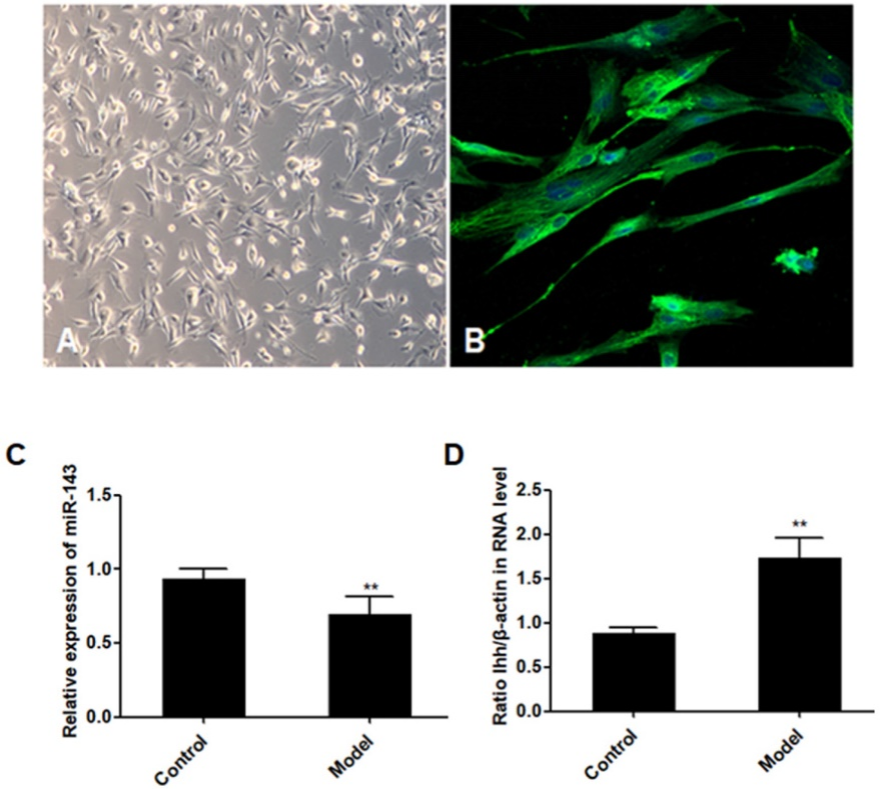
** Aberrant expression of *miR-143* and *IHH.***PCSCs were isolated from the neonate rabbits' distal epiphyseal growth plate. PCSCs were observed with light microscopy (A, magnification 10×) and immunostaining with FGFR-3 (B, magnification 200×). Magnification 400x. In addition, expression of *miR-143* (C) and *IHH* (D) was detected using real time PCR in control and injured growth plates (model group). Experiments were repeated three times with at least three replicates in each assay. Mean values were compared using Student's t-test. *P < 0.05, **P <0.01.

**Figure 2:**
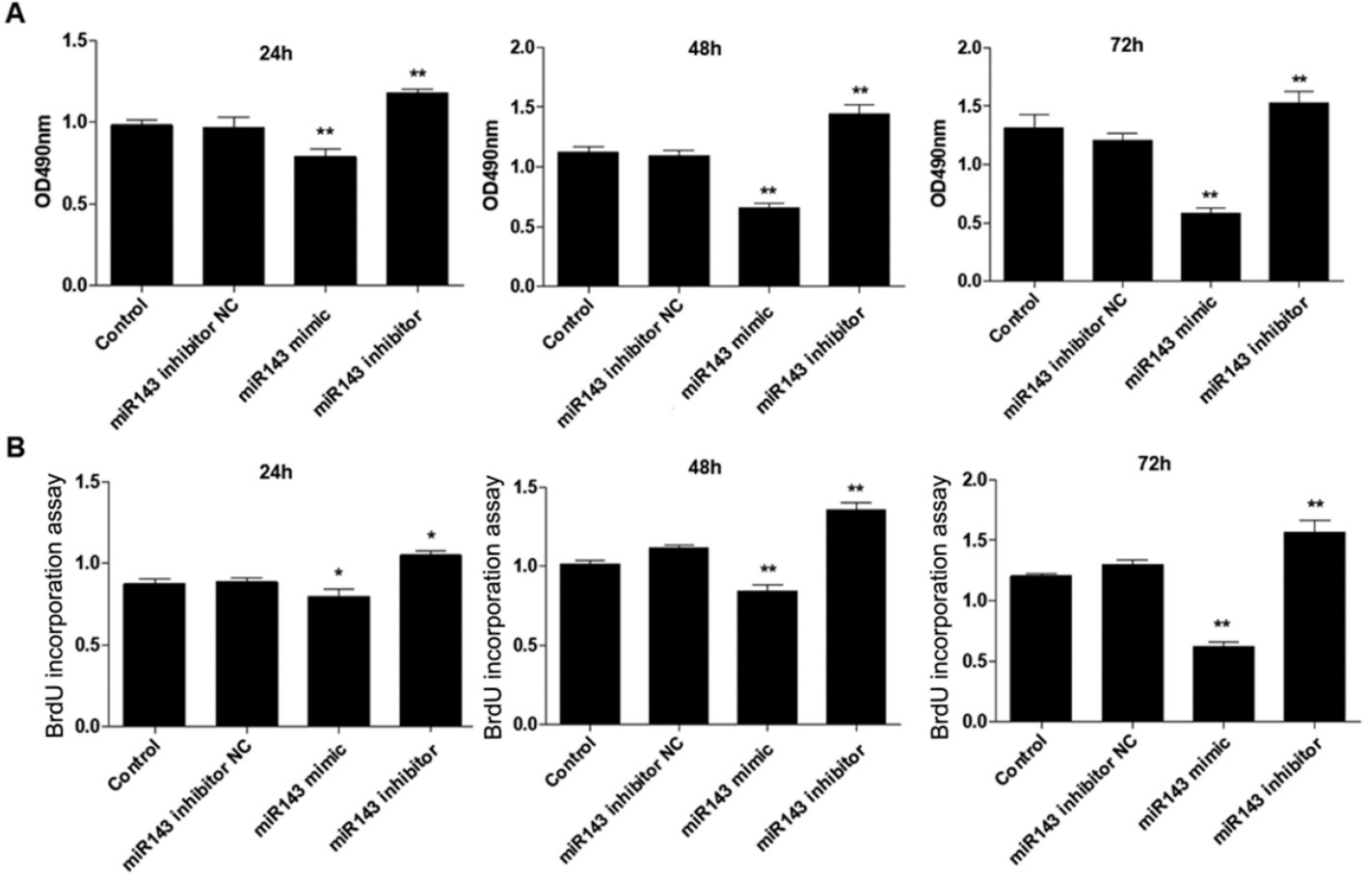
** Effect of miR-143 on growth and DNA synthesis of PCSCs.** PCSCs were transfected with *miR-143* mimic, *miR-143* inhibitor, and negative control (NC). MTT assay (A) and BrdU assay (B) were performed to measure cell viability and DNA synthesis of PCSCs at 24 h, 48 h, and 72 h. Experiments were repeated three times with at least three replicates for each assay. Mean values of multiple groups were compared using one-way ANOVA and Bonferroni post hoc test. *P < 0.05, **P <0.01.

PCSCs, isolated from the neonate rabbits' distal epiphyseal growth plate, were observed using light microscopy (Fig. 1A). Immunofluorescence revealed expression of fibroblast growth factor receptor-3 (FGFR-3), which serves as a marker for PCSCs (Fig. 1B). In addition, real time PCR revealed that expression of *miR-143* was reduced, while *IHH* levels were elevated in the injured growth plate (model group) (Fig. 1C and D). In order to investigate the role of *miR-143* in cell growth, PCSCs were transfected with either *miR-143* mimics or *miR-143* inhibitor and their controls. After culturing for 24, 48, and 72 h, we found that transfection with *miR-143* mimics for 24 h slightly inhibited PCSC growth, while the *miR-143* inhibitor induced PCSC growth (Fig. 2A). At 48 h and 72 h, *miR-143* further suppressed cell growth of PCSCs. In contrast, inhibition of *miR-143* promoted PCSC growth (Fig. 2A). Furthermore, the antiproliferative role of *miR-143* in PCSCs was confirmed with BrdU assay. Results showed that transfection with *miR-143* mimics inhibited DNA synthesis of PCSCs in a time-dependent manner (Fig. 2B), which was enhanced after transfection with the *miR-143* inhibitor at different time points (Fig. 2B). Collectively, these data suggest that *miR-143* may play a negative role in PCSC proliferation.

### *miR-143* regulates apoptotic death and chondrogenesis-related genes in PCSCs

**Figure 3:**
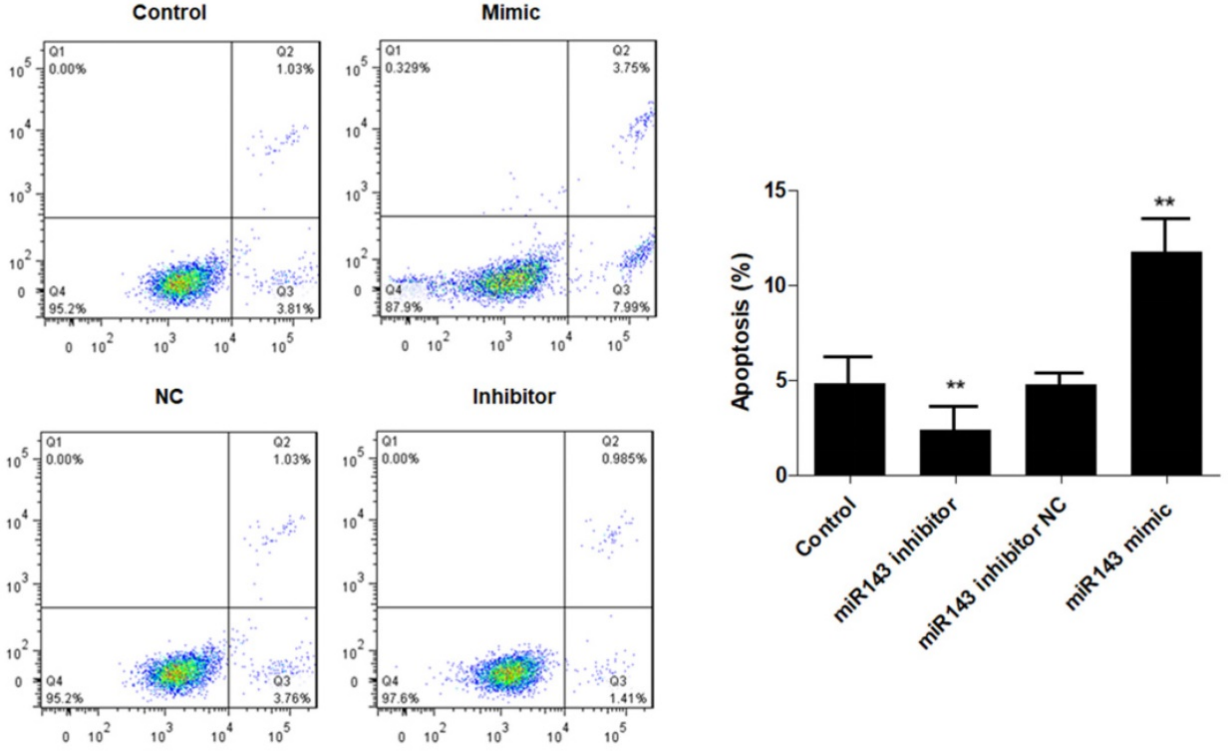
** Effect of *miR-143* on PCSCs apoptosis.** PCSCs were transfected with *miR-143* mimic, *miR-143* inhibitor, and negative control (NC). Flow cytometric analysis was performed to measure cell apoptosis of PCSCs. Experiments were repeated three times with at least three replicates in each assay. Mean values of multiple groups were compared using one-way ANOVA and Bonferroni post hoc test. *P <0.05, **P <0.01.

**Figure 4:**
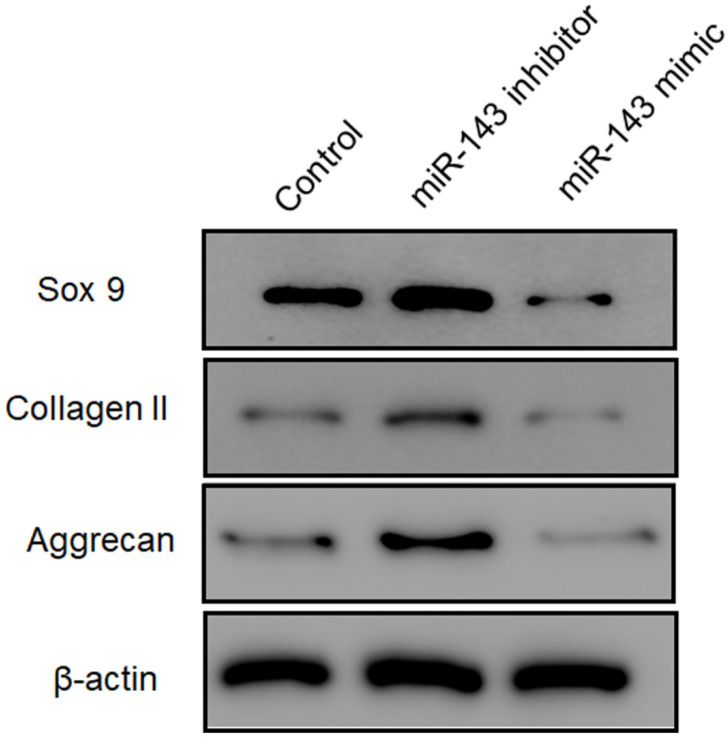
** Effect of *miR-143* on chondrogenesis-related molecules in PCSCs.** PCSCs were transfected with the *miR-143* mimic and the miR-143 inhibitor and protein levels of chondrogenesis-related genes, including Sox9 and collagen II, were determined using western blot. *P <0.05, **P <0.01.

Furthermore, flow cytometry analysis was performed to determine the effect of *miR-143* on PCSC apoptosis. Our data showed that overexpression of *miR-143* increased the percentage of apoptotic PCSCs (11.2±2.2%) compared to the control group (4.5±1.3%). In contrast, downregulation of *miR-143* decreased the percent of apoptotic PCSCs (2.1±1.1%) (Fig. 3). Taken together, these findings revealed that inhibition of *miR-143* may promote PCSC growth via inhibition of apoptosis. In addition, we explored the potential role of *miR-143* in PCSC differentiation. We found that the protein levels of chondrogenesis-related genes, including Sox9, collagen II, and aggrecan, were significantly upregulated after transfection with the *miR-143* inhibitor but were decreased after overexpression of *miR-143* in primary cultured PCSCs (Fig. 4). These data suggested that *miR-143* regulated apoptotic death and chondrogenesis in PCSCs.

### *IHH* is a direct target of *miR-143*

**Figure 5:**
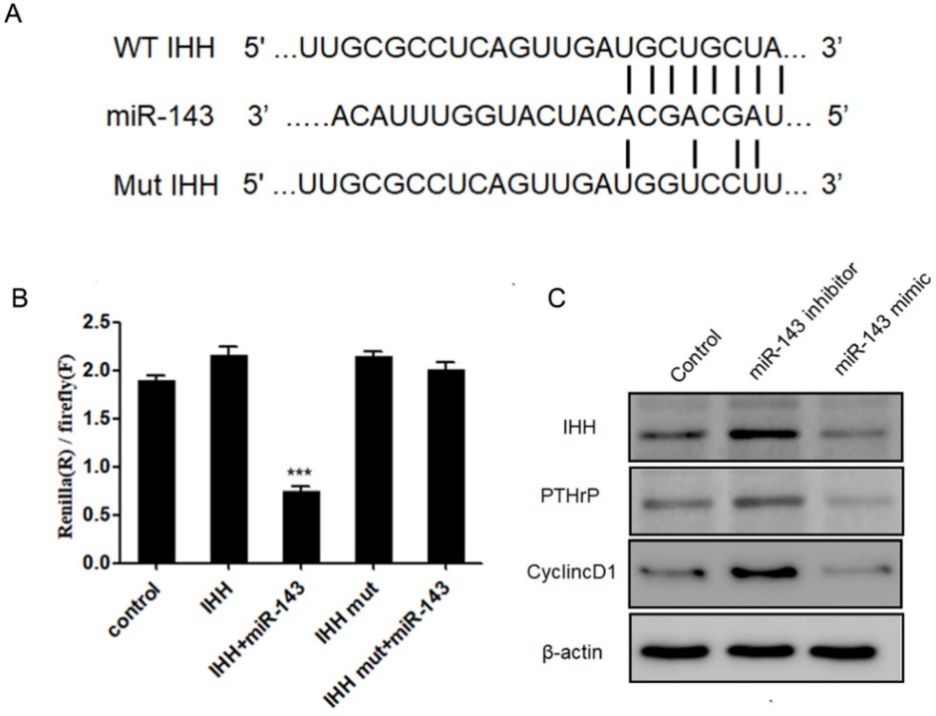
***IHH* is a target of *miR-143.***(A) Schematic diagram of the wild-type and mutant *IHH*. (B) Luciferase reporter assay was used to validate that *IHH* is a direct target of *miR-143*. (C) Protein levels of IHH, PTHrP, and Cyclin D1 were detected with western blot. Experiments were repeated three times with at least three replicates in each assay. Mean values of multiple groups were compared using one-way ANOVA and Bonferroni post hoc test. *P < 0.05, **P <0.01.

Next, we aimed to identify the potential targets of *miR-143* with bioinformatic analysis using the miRNA database (http://www.mirbase.org/), miRanda (http://www.microrna.org), and TargetScan (http://www.targetscan.org/). Our data revealed that the 3'-UTR of *IHH* gene contained conserved binding sites for *miR-143* (Fig. 5A). To validate that *IHH* is a target of *miR-143*, luciferase reporters that contained the wild-type 3'-UTR of *IHH* or a 3'-UTR with a *miR-143* mutated biding site were generated. Consequently, the activity of wild-type *IHH*-3'-UTR was significantly decreased to less than half compared to control in PCSCs co-transfected with *miR-143* and wild-type IHH, but remained unchanged in PCSCs transfected with the mutated *IHH*-3'-UTR (Fig. 5B). Moreover, the protein levels of IHH were suppressed following treatment with *miR-143* mimics but were elevated by two-fold after addition of a *miR-143* inhibitor (Fig. 5C). Furthermore, overexpression of *miR-143* also suppressed PTHrP and Cyclin D1, which were obviously elevated in *miR-143* inhibitor-treated cells (Fig. 5C). Collectively, these findings showed that *IHH* was a direct target gene of *miR-143* in PCSCs.

### *miR-143* mediates PCSC growth and apoptosis by targeting *IHH*

**Figure 6:**
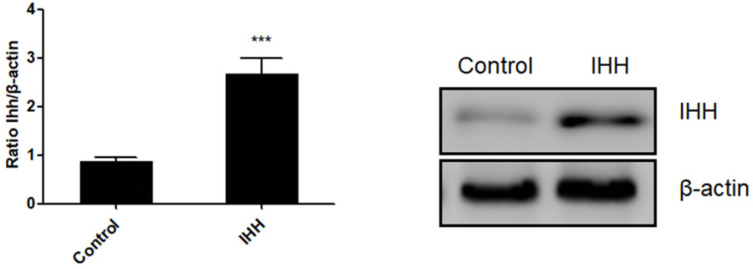
** Ectopic expression of IHH in PCSCs.** PCSCs were transfected with pcDNA3.1(+)-IHH and control plasmid. Forty-eight hours later, total RNA and protein were harvested. The mRNA and protein expression of IHH was determined with real time PCR and western blot, respectively. Experiments were repeated three times with at least three replicates in each assay. Student's t-test. ***P <0.001.

**Figure 7:**
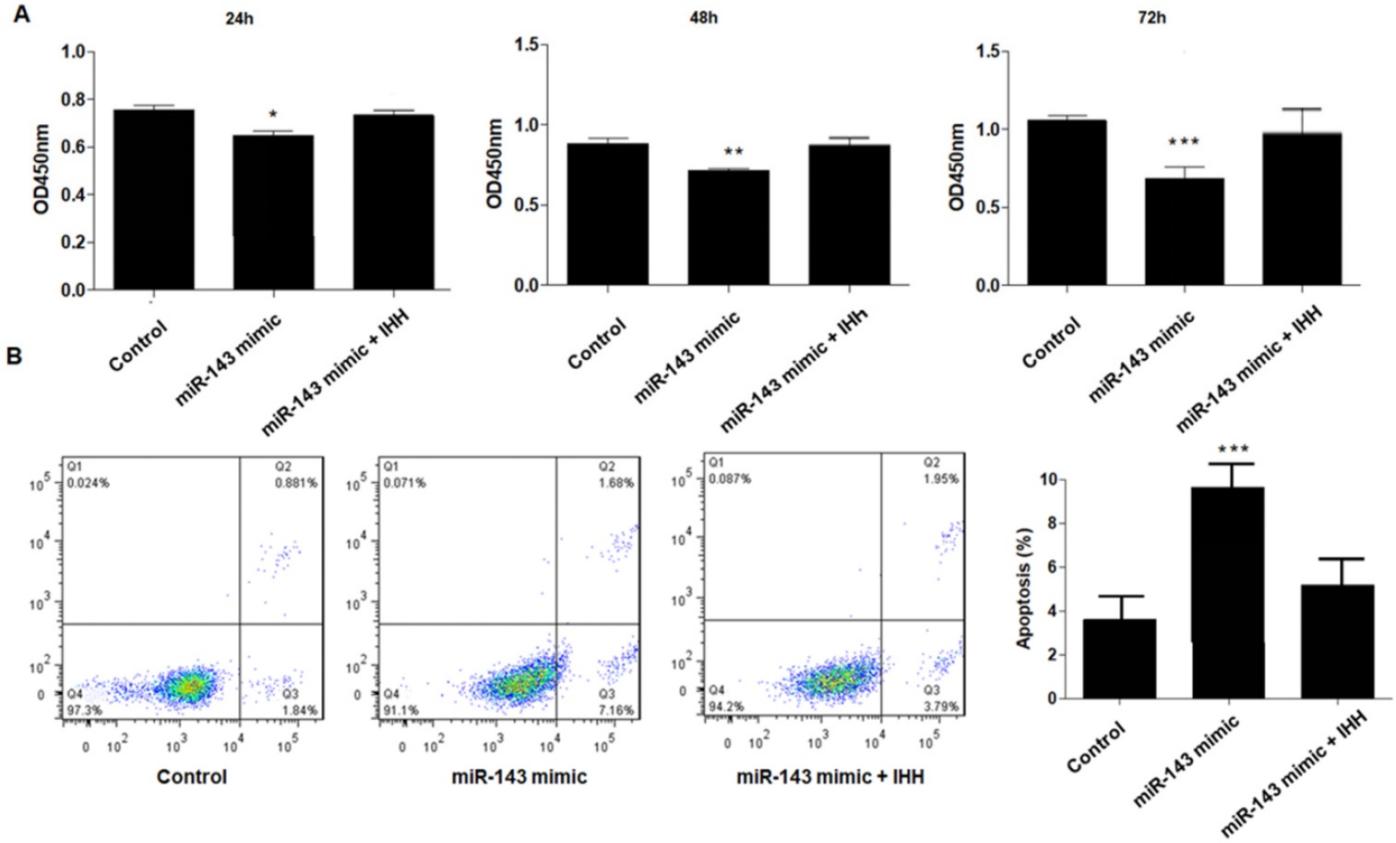
** Upregulation of IHH reverses the effect of *miR-143* on PCSCs.** PCSCs were transfected with *miR-143* mimic alone or pcDNA3.1(+)-IHH followed by MTT assay (A) to measure cell viability of PCSCs at 24 h, 48 h, and 72 h. Moreover, flow cytometry (B) was performed to measure cell apoptosis of PCSCs. Experiments were repeated three times with at least three replicates in each assay. Mean values of multiple groups were compared using one-way ANOVA and Bonferroni post hoc test. *P <0.05, **P <0.01, ***P <0.001.

Moreover, the mechanism of *miR-143* involved in PCSC growth and apoptosis was investigated. In order to validate whether *miR-143* exerts its biological role via IHH, we used the pcDNA3.1 vector to direct expression of IHH. Real time PCR revealed that mRNA levels of *IHH* increased by about three-fold in pcDNA3.1-IHH transfected cells. Consistently, IHH protein levels were significantly elevated in PCSCs transfected with pcDNA3.1-IHH (Fig. 6). Consequently, MTT assay showed that transfection with *miR-143* mimics suppressed PCSC cell viability at 24, 48, and 72 h post-transfection; in contrast, co-transfection with IHH reversed cell viability suppression by *miR-143* in PCSCs (Fig. 7A). Moreover, flow cytometry analysis showed that overexpression of *miR-143* increased the percentage of apoptotic PCSCs, which was reversed after co-transfection with IHH (Fig. 7B).

## Discussion

Our current findings reveal that, by targeting *IHH*, *miR-143* downregulation could promote growth and inhibit apoptosis of PCSCs, thereby modulating chondrogenesis effects. Most of the current studies focus on the regulatory role of *miR-143* in tumorigenesis. For example, *miR-143* may serve as a potential target in osteosarcoma. *In vitro* assays have identified that *miR-143* regulates proliferation, apoptosis, and migration of osteosarcoma cells via targeting multiple key molecules, such as MAPK7 and FOS-Like antigen 2 (FOSL2) 18-20. A growing body of evidence has revealed that miRNAs play essential roles in the regulation of cartilage differentiation and repair 11-14. Gu et al. found that transfection with *miR-155* inhibited osteoblast differentiation by downregulating SMAD5 in mouse preosteoblast cells 21. Zhang et al. demonstrated that increased *miR-93-5p* in patients with trauma-induced osteonecrosis of the femoral head (TIONFH) inhibited osteogenic differentiation by targeting BMP2 22. Several publications have suggested that *miR-143* serves as a tumor suppressor and is involved in many biological processes including cell growth, apoptosis, and migration 23,24. However, the biological function of *miR-143* in PCSCs remains unknown. In the current study, the expression of *miR-143* was significantly downregulated in a rabbit model of growth plate injury, together with elevated levels of IHH. Moreover, *miR-143* expression was negatively correlated with IHH expression. To investigate the biological role of *miR-143*, we transfected either *miR-143* mimics or an inhibitor into PCSCs. As a result, we found that knockdown of *miR-143* significantly increased proliferation and DNA synthesis, and inhibited apoptosis in PCSCs. Conversely, upregulation of *miR-143* decreased cell growth and DNA synthesis and promoted apoptosis in PCSCs. Given the association between apoptosis and proliferation 25, we evaluated the levels of apoptosis in PCSCs overexpressing or downregulating *miR-143*. Our data showed that knockdown of *miR-143* inhibited apoptosis but overexpression of *miR-143* enhanced apoptosis in PCSCs. These findings reveal that antiproliferative function of *miR-143* in PCSCs is mediated by inducing apoptosis. Furthermore, we tested the potential role of *miR-143* in PCSC differentiation. Data showed that protein expression of chondrogenesis-related genes, including Sox9 and collagen II, was significantly downregulated after *miR-143* mimic treatment and elevated in *miR-143* inhibitor-treated PCSCs, suggesting that *miR-143* suppresses chondrogenic differentiation of PCSCs.

The Hh signaling pathway is important for bone development, homeostasis, and repair 26. IHH is a transmembrane protein that forms a receptor complex for Shh together with Smoothened 27. Based on bioinformatic analyses and anti-correlated expression of *miR-143* and IHH, we hypothesized that *miR-143* probably targeted the *IHH* gene to control PCSC growth. As expected, we identified a putative binding site of *miR-143* in the 3'‑UTR region of *IHH*. To validate the direct interaction between *miR-143* and *IHH*, we mutated the putative *miR-143* binding sites at the 3′-UTR of IHH. Dual luciferase reporter assay revealed that the activity of wild-type *IHH*-3'-UTR significantly decreased in PCSCs co-transfected with *miR-143*. In contrast, the dual luciferase activity following co-transfection of the mutant 3'-UTR of *IHH* with *miR-143* mimics compared with co-transfection with control plasmid showed no significant difference, indicating a direct interaction between *miR-143* and the *IHH* gene. Data showed that levels of IHH were suppressed following treatment with *miR-143* mimics but were elevated after addition of *miR-143* inhibitor. IHH, which is required for the synthesis of PTHrP, interacts with PTHrP in a negative feedback loop to regulate chondrocyte differentiation 28. Consequently, levels of PTHrP and IHH-targeted Cyclin D1 were suppressed following treatment with *miR-143* mimics but were elevated after addition of a *miR-143* inhibitor, showing that *miR-143* inhibits the IHH pathway in PCSCs.

Most bones develop through the process of bone formation, and cartilage tissues are formed through condensation of mesenchymal cells, followed by differentiation into chondrocytes. IHH, as a member of the hedgehog family, is expressed in prehypertrophic and early hypertrophic chondrocytes 29. IHH regulates the interaction between mesenchymal stem cells and osteoclasts for bone homeostasis and repair. Moreover, IHH stimulates precartilaginous stem cell proliferation and inhibits osteoblast development in endochondral bones 30. In the present work, IHH was directly targeted by *miR-143* and its expression was found to be negatively correlated with that of *miR-143*. Therefore, *miR-143* may regulate the apoptosis and growth in PCSCs through targeting IHH. As expected, co-transfection with miR-143 mimics and IHH reversed the suppressive role of *miR-143* in PCSCs. In addition, overexpression of *miR-143* promoted apoptosis in PCSCs, which was reversed by co-transfection with IHH. Collectively, these findings determine that the effect of *miR-143* overexpression was neutralized by IHH overexpression.

In conclusion, our study demonstrated that *miR-143* regulated growth and apoptosis in PCSCs. Moreover, the current study revealed that *miR-143* directly targeted the *IHH* gene and inhibited its endogenous expression at both the mRNA and protein levels. These findings suggest that *miR-143* may serve as a novel target for cartilage regeneration and repair.
